# Corona virus vaccine hesitancy among higher education students in Adama City, Oromia, Ethiopia

**DOI:** 10.3389/fpubh.2024.1364225

**Published:** 2024-03-25

**Authors:** Dawit Abebe, Tewodros Mengistu, Enku Afework Demssie, Sinetibeb Mesfin

**Affiliations:** ^1^School of Nursing and Midwifery, College of Health and Medical Sciences, Jigjiga University, Jigjiga, Ethiopia; ^2^Department of Public Health, Adama General Hospital and Medical College, Adama, Ethiopia; ^3^School of Nursing and Midwifery, College of Health and Medical Sciences, Haramaya University, Harar, Ethiopia

**Keywords:** COVID-19, vaccine hesitancy, higher education institutions, Ethiopia, Adama City

## Abstract

**Background:**

Vaccination stands as the most efficient approach for managing the continued transmission of infections and preventing the emergence of novel variants. Coronavirus disease 2019 (COVID-19) vaccine hesitancy poses a significant burden in the fight to achieve herd immunity.

**Methods:**

A cross-sectional study, based on institutional parameters, was conducted among a cohort of 530 higher education students, selected via a simple random sampling method. Study participants were selected using a systematic random sampling technique from February to March 2022. Structured questionnaire data were gathered and subsequently analyzed using SPSS version 21. The strength of the association between various factors and COVID-19 vaccine hesitancy was assessed using the odds ratio along with its 95% confidence interval. Statistical significance was deemed to be present at a *p*-value of < 0.05.

**Result:**

The prevalence of coronavirus vaccine hesitancy was 47.5%. The factors that were found to be significantly associated with COVID-19 vaccine hesitancy were residential address (AOR = 2.398, 95% CI: 1.476–3.896); agreeing with leaders and groups that do not support COVID-19 vaccination (AOR = 2.292, 95% CI: 1.418–3.704); coming from a community whose leaders support COVID-19 vaccination for young adults (AOR = 0.598, 95% CI: 0.381–0.940), and believing that COVID-19 vaccines are safe (AOR = 0.343,95% CI: 0.168–0.701).

**Conclusion:**

Approximately five out of 10 students who participated in this study were hesitant to get vaccinated against coronavirus. Incorporating messages and initiatives into local plans to specifically target the factors identified in this study is imperative for substantially increasing the COVID-19 vaccine uptake among students in higher education institutions.

## Background

Coronavirus disease 2019 (COVID-19) is an infectious disease caused by the SARS-CoV-2 virus (1). Originating in Wuhan, China, COVID-19 swiftly disseminated, prompting the World Health Organization to officially declare it as a pandemic on 11 March 2020. As of 16 October 2021, a total of 240,278,867 confirmed cases and 4,892,799 deaths were reported worldwide, while there are more than 362,000 confirmed cases and 6,300 deaths registered in Ethiopia (1).

The Strategic Advisory Group of Experts on Immunization (SAGE) working group, instituted in 2012, delineated vaccine hesitancy as the postponement or outright rejection of vaccination, notwithstanding the accessibility of vaccination services (2). In the year 2019, predating the advent of the COVID-19 pandemic, the World Health Organization identified vaccine hesitancy as one of the 10 worldwide threats to public health (3). Vaccines stand as the sole clinical preventive measure for managing infection and mortality attributed to COVID-19; however, based on worldwide reports, vaccine hesitancy and failure to get vaccinated may pose a significant threat in the fight against the pandemic (2, 4, 5).

Vaccine hesitancy manifests as a multifaceted and context-dependent phenomenon, exhibiting variations over time, location, and with different vaccines. This phenomenon is shaped by factors such as complacency, convenience, and confidence (6). It can be associated with individual convictions, incentives, cognitive acumen, and the level of awareness (7). Moreover, the exchange of information between healthcare providers and vaccine recipients plays a pivotal role in arriving at a shared decision regarding vaccination (8).

Vaccine hesitancy exhibits significant variations across countries, with the prevalence of vaccine hesitancy ranging from 6.4 to 61.8% (9). In sub-Saharan Africa, this hesitancy is even more pronounced, ranging from 14 to 76% (10, 11). In Ethiopia, varying levels of vaccine hesitancy have been reported, primarily among healthcare staff (3, 6, 7). Understanding the extent of vaccine hesitancy among higher education students will be crucial for Ethiopia to achieve its planned herd immunity goals across the country.

On 13 March 2021, Ethiopia launched its vaccination campaign, prioritizing frontline health professionals and older adults. By October 2021, the nation had successfully administered vaccinations to 3.5 million people (11).

## Materials and methods

### Study setting, design, and period

An institutional-based cross-sectional study was conducted at eight higher education institutions found in Adama City, which is situated 100 km southeast of the capital of Ethiopia, Addis Ababa. Of the total population, ~86,069 are young adults attending higher education institutions. In the city administration, there are eight higher education institutions, four universities (Adama Science and Technology University, Harambe University, Rift Valley University, and Unity University), and four colleges (Adama TVET College, Adama Comprehensive Hospital and College of Health Sciences, Oromia Police college, and Adama General Hospital and Medical college). The study was conducted between 01 February and 30 March 2022.

### Source population, study population, and eligibility criteria

The source population comprised students enrolled in higher education institutions within the city during the study period, and the study population encompassed all students from these institutions who provided consent to participate in the study. Individuals who refused to participate were excluded from the study.

### Sample size determination and sampling procedure

The sample size was determined using EPI Info version 7 statistical software, employing the double population proportion formula, with COVID-19 vaccine distrust as an independent variable (8). The considerations included a 95% confidence level, 80% power, a 1:1 ratio, and an additional 10% contingency to account for non-response rates, finally resulting in the recruitment of 530 study participants. Four higher educational institutions were selected through simple random sampling. Study participants were selected from those who were attending class during the data collection period from February to March 2022 using a systematic random sampling technique. The sampling interval (*K*) was calculated by dividing the total number of students who were attending classes in the higher education facility during the data collection period by the sample size. A number between one and *K* was taken as a random start, and every *K*th value was selected as a sample unit. The sample size was allocated for each higher education facility proportionally, which is 132 for three institutions and 134 for one institution.

### Data collection tools and procedure

#### Data collection instrument

Data were collected through the utilization of a structured, pre-tested, and self-administered questionnaire in English. These meticulously designed questionnaires encompass various sections. **Part I** focuses on the sociodemographic variables of the participant. **Part II** emphasizes questions related to assessing contextual influences of COVID-19 vaccine hesitancy. **Part III** examines questions related to assessing individual and group influences of COVID-19 vaccine hesitancy. **Part IV** focuses on questions related to assessing vaccine/vaccination-specific issues of COVID-19 vaccine hesitancy. The questionnaires were extracted and adapted from different kinds of valid and reliable instruments used in previous literature on the same topics (4, 12, 13).

#### Data collection procedure

Four health officers, possessing prior experience in data collection, were tasked with the responsibility of gathering data. To supervise both the data collectors and the entire data collection process, two public health experts were selected. The principal investigator dedicated a day to providing training for the supervisor and data collectors, covering the study's objectives, the content of the instruments, the participant selection process, how to fill out the questionnaire, and how to approach individuals ethically. All study participants had their understanding of the study's objective, the consent form, the confidentiality issue, and informed consent guaranteed. The total activity during the data collection period was strictly supervised by the principal investigator and supervisors.

### Variables of the study

**Dependent variable:** COVID-19 Vaccine hesitancy

**Independent variables: Socio demographic factors** (Age, gender, socioeconomic status, Level of education, geographic area of origin, ethnic group, and Religion).

**Contextual influences**- communication and media environment, anti- or pro-vaccination lobbies, historical influences, politics/policies and perception of the pharmaceutical industry.

**Individual and group influence related factors**- knowledge, health system and healthcare providers trust, personal experience and risk/benefit (perceived, heuristic).

#### Operational definitions

**COVID-19 vaccine hesitancy:** this is characterized as a postponement in acceptance or an outright refusal of vaccination, even in the presence of available vaccination services (14).

**Higher education:** any form of education provided in tertiary institutions, typically culminating in the conferment of a designated degree, diploma, or advanced certificate upon the completion of a course of study.

### Data quality control

A half-day training session was conducted for both data collectors and supervisors, focusing on elucidating the study's objectives and significance, instructing on the adept collection of pertinent information, detailing the procedures of data collection techniques, and thoroughly covering the questionnaire's contents. The questionnaire underwent a pre-testing phase involving a representative sample of 27 students (5% of the sample size) from Admas University, and this institution was not included during the major data collection for the research. A necessary modification was made to the questionnaire based on the nature of the identified gaps. Data were entered into Epi-data version 7.2.1 and cleaned and explored for outliers, missed values, and any inconsistencies.

### Data processing and analysis

After checking, coding, and entering Epi data version 3.1 and validating and comparing it to the original data, corrective measures were taken accordingly. Data were exported to the Statistical Package for Social Science [SPSS] Version-27 software for analysis. Through SPSS's transform function, variables were computed and recorded. The study's outcome variable, COVID-19 vaccine hesitancy, was measured by asking participants the following questions: “Will you get vaccinated if you get COVID-19 vaccine?”, for which the answer was dichotomized into “Yes” or “No” alternatives. Students who responded “Yes” were recorded as COVID-19 vaccine acceptance, while those who responded “No” were coded as COVID-19 vaccine hesitancy.

A descriptive analysis utilizing frequency tables, proportions with a 95% confidence interval, and summary measures was conducted. To choose candidate variables for multivariable analysis, a bivariable logistic regression analysis was applied. Based on the presumption of selection criteria, variables with a *p*-value of < 0.25 were taken as candidates for the final multivariable analysis model. The multi-collinearity test was carried out to observe the linear correlation among independent variables by using standard error. The Hosmer-Lemeshow goodness of fitness test was used to assess the appropriateness of the model; the result was negligible (*p* = 0.65), indicating that the model was fitted. Using adjusted odds ratio (AOR) with 95% confidence intervals (95% CI), the strength of associations between the dependent and independent variables was finally evaluated. A *p*-value of < 0.05 was used to indicate the significance of the association.

## Results

### Sociodemographic characteristics of respondents

The median age of those 530 higher education students who were selected for the study was 22 years (IQR = 2). Only 17 (3.2%) of the selected students were 30 years of age and above. A greater proportion of the student population, that is, 377 (71.1%), were male, and most of the study participants, 485 (91.5%), were single. The majority of the students belong to the Oromo and Amhara ethnic groups, and 377 (71.1%) students were urban residents. More than half of the participants (52.6%) were Orthodox Christian by religion, and 471 (88.9%) students were attending their bachelor's degree in their respective higher education institutions. Most of them, 294 (55.5%), reported that they came from low-monthly income families, followed by 161 of them (30.4%) from middle-monthly income families (Table 1).

**Table 1:** Socio-demographic characteristics of higher education students in Adama City, Oromia, Ethiopia, 2022 (n = 530).

**Variables**	**Frequency (*n*)**	**Percent (100%)**
**Age group (in years)**		
= < 20	40	7.5
21–25	403	76
26–30	70	13.2
= >35	17	3.2
**Sex**		
Female	135	28.9
Male	377	71.1
**Ethnicity**		
Oromo	205	38.7
Amhara	165	31.1
Tigre	20	3.8
Gurage	48	9.1
Other	92	17.4
**Residence**		
Urban	377	71.1
Rural	153	28.9
**Educational level**		
College diploma	2	0.4
Degree	471	88.9
Masters and above	57	10.3
**Relationship status**		
Single	485	91.5
Married	38	7.2
Separated	7	1.3
**Estimated family income**		
Low	294	55.5
Middle	161	30.4
High	75	14.2

### Contextual influences of COVID-19 vaccine hesitancy

The majority of the study participants, 334 (63%), trust health professionals for information regarding the COVID-19 vaccine, while social media is the least trusted source, with 209 (39.4%) students indicating their trust in it for information about the COVID-19 vaccine. Among all the study participants, 270 (50.9%) students re-considered the choice to get vaccinated after hearing or reading reports about the COVID-19 vaccine on social media. A large proportion of students, 345 (65.1%), agree with some groups or leaders that do not support COVID-19 vaccination for different reasons. Whereas, leaders (religious, political, teachers, and healthcare workers) supporting COVID-19 vaccines for young adults in their respective communities are 52.3% (Table 2).

**Table 2:** Contextual influences of COVID-19 vaccine hesitancy among higher education students in Adama City, Oromia, Ethiopia, 2022 (n = 530).

**Variables**	**Frequency (*n*)**	**Percent (100%)**
**Trust most information**		
Government sources	67	12.6
Social media	52	9.8
Health professionals	334	63
Family/peer groups	62	11.7
Pharmaceutical industries	15	2.8
**Trust least information**		
Government sources	146	27.5
Social media	209	39.4
Health professionals	19	3.6
Family/peer groups	84	15.8
Pharmaceutical industries	72	13.6
**Re-consideration the info from social media**		
No	260	49.1
Yes	270	50.9
**Agree with some groups or leaders that do not support COVID-19 vaccination**		
No	185	34.9
Yes	345	65.1
**leaders support COVID-19 vaccination**		
No	253	47.7
Yes	277	52.3
**Event in the past discourage vaccination**		
No	296	55.8
Yes	234	44.2
**Government trust in making best decision in the interest of the people**		
No	334	64.9
Yes	186	35.1
**Trust pharmaceutical industries in provision of safe vaccines**		
No	374	70.6
Yes	156	29.4
**Think government is pushed by lobbyists**		
No	190	35.8
Yes	340	64.2
**Know what is COVID-19 vaccine**		
No	100	18.9
Yes	430	81.1
**Know COVID-19 vaccine does to your body**		
No	184	34.7
Yes	346	65.3
**Feel that you know which COVID-19 vaccine you get**		
No	399	75.3
Yes	131	24.7
**Think it's important to get vaccinated to protect others that can't**		
No	270	50.9
Yes	260	49.1
**You or someone you know had bad reaction to vaccine**		
No	220	41.5
Yes	309	58.3
**Felt health professionals or government pushing you vs. your will to get vaccine**		
No	267	50.4
Yes	263	49.6
**You will get vaccine when available**		
Yes	50	9.4
Maybe	228	43
Never	252	47.5
**Do you agree with vaccine mandate?**		
No	401	75.7
You	129	24.3
**Believe COVID-19 vaccine is safe**		
No	430	81.1
Yes	100	18.9

### COVID-19 vaccine hesitancy

**Figure 1:**
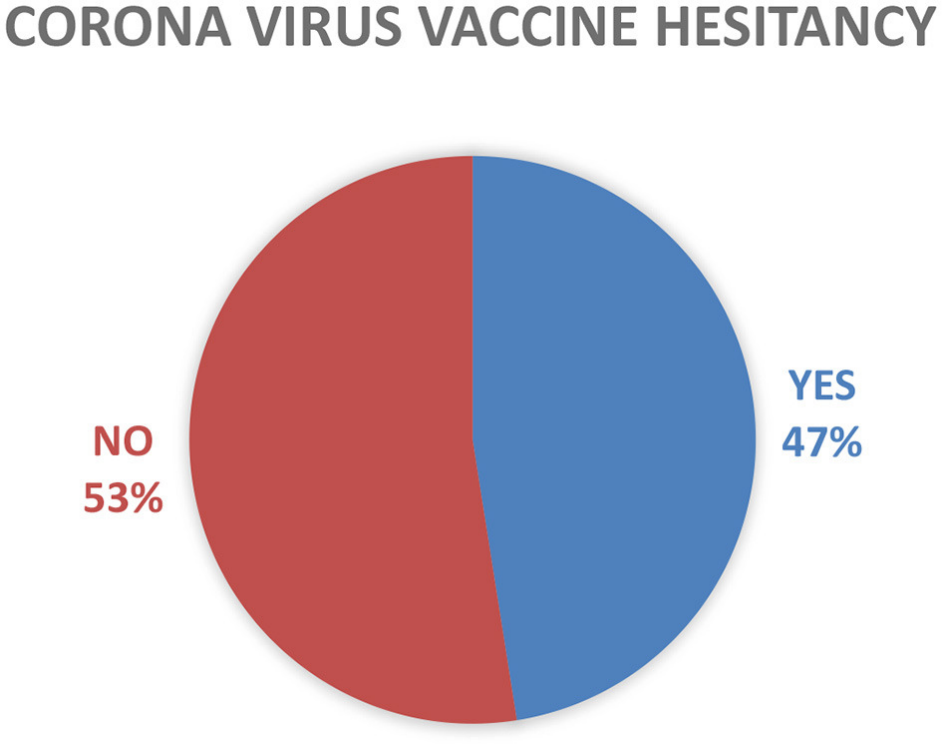
Categorized reported of vaccine hesitancy among higher education students in Adama City, Oromia, Ethiopia, 2022 (*N* = 530).

COVID-19 vaccine hesitancy was measured by asking participants, “Will you get vaccinated if you get COVID-19 vaccine?”, for which the answer was dichotomized into “Yes” or “No”. The study showed that the prevalence of coronavirus vaccine hesitancy among students in higher education institutions in Adama City administration is 47.5% (Figure 1).

### Factors associated with COVID-19 vaccine hesitancy

A total of 8 variables, including residence in rural areas [COR = 1.632 (95% 1.117, 2.382)]; agreeing with leaders and groups that do not support COVID-19 vaccination [COR = 3.814 (2.585, 5.626)]; a previous history of bad reaction to the vaccine [COR = 1.740 (1.225, 2.470)]; coming from a community whose leaders support COVID-19 vaccination for young adults [COR = 0.388 (0.273, 0.550)]; thinking it is important to get vaccinated to protect those that cannot be vaccinated for different reasons [COR = 0.166 (0.114, 0.241]; believing COVID-19 vaccines are safe [COR = 0.131 (0.072, 0.238)]; trusting their government in making the best decision in the interest of its people [COR = 0.391 (0.269, 0.567)]; and thinking the government is pushed by lobbyists or vaccine makers to recommend certain vaccines [COR = 1.045 (0.732, 1.491)], were found to have a statistically significant association with coronavirus hesitancy.

To control confounding and find independent factors of coronavirus vaccine hesitancy, multivariable analysis was used. Variables with a *p*-value of < 0.25 in the bi-variable logistic regression analysis were candidates for multivariable analysis. Out of those associated variables in the bi-variable analysis, the odds of vaccine hesitancy were 2.4 times higher in students who were from rural areas compared to their urban counterparts (AOR = 2.398, 95% CI: 1.476–3.896). The odds of vaccine hesitancy were 2.3 times higher in students who agree with leaders and groups that do not support COVID-19 vaccination compared to those who support the vaccine (AOR = 2.292, 95% CI: 1.418–3.704). Vaccine hesitancy was 40.2% less likely in students who came from a community whose leaders supported COVID-19 vaccination for young adults compared to those whose leaders did not support it (AOR = 0.598, 95% CI: 0.381–0.940). The odds of vaccine hesitancy were 65.7% less likely in those who believed COVID-19 vaccines were safe compared to those who did not (AOR = 0.343, 95% CI: 0.168–0.701) (Table 3).

**Table 3:** Bivariate and multivariate logistic regression analysis for factors associated with Corona virus vaccine hesitancy among students in higher education institutions in Adama city, Oromia Ethiopia 2022 (N = 530).

**Variable**		**Vaccine hesitant**		**Bivariate logistic regression (COR)**	**Multivariate logistic regression AOR (with 95% CI)**
		**Yes (*****N*** = **252)**	**No (*****N*** = **278)**		
Residential address	Urban	166	211	1	1
	Rural	86	67	1.632	**2.398 (1.476, 3.896)** ^*^
Agree with leaders or groups that do not support COVID-19 vaccine vaccination	No	50	135	1	1
	Yes	202	143	3.814	**2.292 (1.418, 3.704)** ^*^
Previous history of bad reaction to vaccine	No	87	133	1	1
	Yes	165	145	1.740	1.751 (0.119, 2.738)
Being from a community whose leaders support COVID-19 vaccination for young adults	No	151	102	1	1
	Yes	101	176	0.388	**0.598 (0.381, 0.940)** ^**^
Government trust in making best decision in the interest of the people	No	191	153	1	1
	Yes	61	125	0.391	0.557 (0.336, 1.90)
Think it is important to get vaccinated to protect those that can't be vaccinated for different reasons	No	184	86	1	1
	Yes	68	192	0.166	0.299 (0.191, 1.469)
Believe COVID-19 vaccines are safe	No	238	192	1	1
	Yes	14	86	0.131	**0.343 (0.168, 0.701)** ^*^
Think government is pushed by lobbyists or vaccine makers to recommend certain vaccines	No	89	101	1	1
	Yes	163	177	1.045	0.519 (0.327, 1.826)

* *P*-value < 0.05,

** *P*-value < 0.005.

CI, confidence interval; COR, crude odds ratio; AOR, adjusted odds ratio.

## Discussion

The study has provided insights into factors that determine vaccine hesitancy and its prevalence in higher education students in Adama City. The prevalence of coronavirus vaccine hesitancy in this study was 47.5%, which is comparatively low to a study conducted at Gondar University in northern Ethiopia (73%) (15). However, the prevalence is higher than that reported in Nevada (16) and Bangladesh (29.8%) (17). These differences are mainly due to study period differences and the timing of COVID-19 vaccine distribution in different parts of the world given the fact that attitudes toward vaccinations have been shown to change over time. Contextual factors, such as cultural beliefs, access to healthcare, government messaging, and socioeconomic conditions, which vary significantly between regions, can also contribute to differences in vaccine hesitancy rates.

The most frequently stated reasons for those hesitating vaccination were a lack of trust due to the fast vaccine development process, 71 (31.8%); a desire to wait and see what happens to vaccinated people, 69 (30.9%); and fear of adverse effects, 42 (18.8%). This finding is supported in the study conducted at Jimma University where study participants claim related concerns (29). These issues can be resolved by public education on vaccine testing, Food and Drug Administration authorization and approval procedures, and side effect studies, all of which will increase the acceptance of the COVID-19 vaccine.

In this study, it was shown that several independent variables had statistically significant relationships with the outcome variable. Vaccine hesitation is more likely observed among rural residents. This finding is in line with the study findings conducted in Ethiopia and India (18, 19), which might be due to rural populations not having easy access to medical professionals who can respond to their questions and alleviate their concerns about the vaccine. Additionally, due to the lack of healthcare infrastructure and resources, vaccination distribution and administration may be more difficult in rural areas.

Students who agree with leaders or groups that do not support COVID-19 vaccination have a high chance of hesitancy. This finding is consistent with the findings of the study conducted in Malaysia (20). A student may be influenced by the ideals and views of a community or group they are a member of if that community or group opposes COVID-19 vaccinations. Although students may disagree with or have reservations about vaccination, they could feel pressure to adhere to the group's norms and views, in contrast to students from a community whose leaders support COVID-19 vaccination for young adults, which is negatively associated with hesitancy. This finding can be explained by the fact that students are more likely to receive correct information about the advantages and safety of the COVID-19 vaccine if they belong to a group or community that promotes vaccinations. They might also feel obligated to defend their neighborhood and themselves against the virus, which may result in less hesitation about vaccinations.

Consistent with the study conducted in the Ethiopian general population (21), students who believe COVID-19 vaccines are safe have a negative association with COVID-19 vaccine hesitation. This negative association may be due to the findings that most of the students, 334 (63%), trust health professionals for information regarding the COVID-19 vaccine. In comparison, social media was the least trusted source, as responded by 209 (39.4%) students, which is in line with a study conducted in Lebanon (12), which is due to the fact that they get appropriate information about vaccinations and have trust in the medical and scientific community and believe that the vaccines have undergone rigorous testing and approval processes.

## Data availability statement

The raw data supporting the conclusions of this article will be made available by the authors, without undue reservation.

## Ethics statement

The studies involving humans were approved by Adama General Hospital and Medical College Research Ethics Review Committee (Project Reg. No. RERC 021/2022). The studies were conducted in accordance with the local legislation and institutional requirements. The participants provided their written informed consent to participate in this study.

## Author contributions

DA: Methodology, Project administration, Validation, Formal analysis, Investigation, Writing—original draft, Writing—review & editing. TM: Conceptualization, Data curation, Formal analysis, Funding acquisition, Investigation, Methodology, Project administration, Resources, Supervision, Validation, Visualization, Writing—original draft, Writing—review & editing. ED: Investigation, Methodology, Writing—original draft, Writing—review & editing. SM: Resources, Validation, Visualization, Formal analysis, Methodology, Writing—original draft, Writing—review & editing.
